# Exposure of Ophthalmologists to Patients' Exhaled Droplets in Clinical Practice: A Numerical Simulation of SARS-CoV-2 Exposure Risk

**DOI:** 10.3389/fpubh.2021.725648

**Published:** 2021-09-20

**Authors:** Yanchao Fan, Li Liu, Hui Zhang, Yingping Deng, Yi Wang, Mengjie Duan, Huan Wang, Lixiang Wang, Leifeng Han, Yalin Liu

**Affiliations:** ^1^State Key Laboratory of Green Building in Western China, Xi'an University of Architecture and Technology, Xi'an, China; ^2^School of Building Services Science and Engineering, Xi'an University of Architecture and Technology, Xi'an, China; ^3^Department of Building Science, School of Architecture, Tsinghua University, Beijing, China; ^4^YuanPu EyePro Biopharm Limited, Chengdu, China; ^5^Department of Ophthalmology, West China Hospital, Sichuan University, Chengdu, China; ^6^East China Architectural Design and Research Institute, Shanghai, China

**Keywords:** ophthalmology, inhalation exposure, computational fluid dynamics simulation, breathing mode, relative humidity, SARS-CoV-2

## Abstract

**Background:** Lack of quantification of direct and indirect exposure of ophthalmologists during ophthalmic diagnostic process makes it hard to estimate the infectious risk of aerosol pathogen faced by ophthalmologists at working environment.

**Methods:** Accurate numerical models of thermal manikins and computational fluid dynamics simulations were used to investigate direct (droplet inhalation and mucosal deposition) and indirect exposure (droplets on working equipment) within a half-minute procedure. Three ophthalmic examination or treatment scenarios (direct ophthalmoscopic examination, slit-lamp microscopic examination, and ophthalmic operation) were selected as typical exposure distance, two breathing modes (normal breathing and coughing), three levels of ambient *RH* (40, 70, and 95%) and three initial droplet sizes (50, 70, and 100 μm) were considered as common working environmental condition.

**Results:** The exposure of an ophthalmologist to a patient's expiratory droplets during a direct ophthalmoscopic examination was found to be 95 times that of a person during normal interpersonal interaction at a distance of 1 m and 12.1, 8.8, and 9.7 times that of an ophthalmologist during a slit-lamp microscopic examination, a surgeon during an ophthalmic operation and an assistant during an ophthalmic operation, respectively. The ophthalmologist's direct exposure to droplets when the patient cough-exhaled was ~7.6 times that when the patient breath-exhaled. Compared with high indoor *RH*, direct droplet exposure was higher and indirect droplet exposure was lower when the indoor *RH* was 40%.

**Conclusion:** During the course of performing ophthalmic examinations or treatment, ophthalmologists typically face a high risk of SARS-CoV-2 infection by droplet transmission.

## Introduction

The COVID-19 pandemic by SARS-CoV-2 causes million people infected worldwide every day, and 3 million deaths up to now (1). This fast spread disease transmits people *via* short-airborne droplet inhalation, mucosa deposition and indirect contact (2). Some data showed that healthcare workers (HCWs) have higher proportion of SARS-CoV-2 infection in many countries (3, 4). Certain hospital areas, such as respiratory departments, intensive care units and surgery departments, are identified as higher risk zones (5). HCWs in ophthalmology clinics/hospitals are also a group of high risk population, due to the nature of clinical practice. It could not be ignored that three of the four doctors who died of COVID-19 at the beginning of the pandemic were ophthalmologists. Recent outbreak of SARS-CoV-2 Delta variant in the world revealed that the virus is changing to be more contagious than early SARS-CoV-2 (6, 7). The aerosol transmission of Delta variant between people is more effective as the viral load in the respiratory system of infected peoples is much higher than original SARS-CoV-2 (8). The risk level and risk spots in ophthalmology practice should be evaluated in order to establish effective protections.

Respiratory droplets, which are released during breathing, speaking, coughing or sneezing, can carry pathogen and suspended in the air for various lengths of time, and transmit virus from person to person (9). More evidence suggests that SARS-CoV-2 is primarily transmitted *via* the respiratory droplet route (10, 11), largely by inhalable viral aerosols (12, 13).

Routine ophthalmic examinations and operations that are performed in eye clinics and hospitals occur within the range of droplet and aerosol transmission. Particularly, the face-to-face encounters between ophthalmologists and patients during direct ophthalmoscopic and slit-lamp microscopic examinations and similar procedures directly expose ophthalmologists to patients' exhaled respiratory flows, and thus carry a relatively higher risk of nosocomial transmission of SARS-CoV-2 (14).

As far as now there are very limited data to provide information of respiratory droplets exposure of ophthalmologists during ophthalmic examination or treatment procedures, and thus it is difficult to estimate the SARS-CoV-2 infection risks faced by these clinicians. To address this research gap, this study simulated typical working environment and calculated droplet exposure of ophthalmologists during short time of examination and treatment procedures. The factors such as the patient's breathing mode, relative humidity (RH) and initial droplet size were analyzed as potential influence of magnitude of exposures. The results of the investigation provide scientific data to understand ophthalmologists' risk in transmission of SARS-CoV-2 in their routine work, and valuable data to guide protective measures of ophthalmology clinics/hospitals. While the new mutant variants of SARS-CoV-2 spreads faster in aerosol, ophthalmic HCWs need to keep regular protection in the future to preventing doctor-patient cross-infection. This simulation work demonstrated the high exposure of aerosol pathogen of ophthalmologists and provided data to identify risk zone in eye clinics/hospitals during pandemic period.

## Methodology

### Physical Model

Recently there are investigations using numerical model of thermal manikins and computational fluid dynamics to study the transmission of exhaled pathogen between people in public environment (15, 16). The advantage of this method is that can obtain results quickly with good accuracy at low cost and can be shown intuitively. The limitation is that the turbulence model and boundary conditions are simplified to some extent, and cause some errors in simulate calculation. The uncertainty of the computational model comes from the Discrete Random Walk (DRW) model which employed random method to simulate the influence of instantaneous turbulent velocity fluctuation on particle trajectory in fluid phase flow field.

Hospital ophthalmology clinics differ in size, structure, layout and ventilation mode, and ophthalmic examinations are performed at close range (i.e., with <0.8 m between an ophthalmologist and a patient). Therefore, this study considered the effects of weakened room ventilation and geometric parameters on the local microenvironmental flow field and the exposure of an ophthalmologist to droplets exhaled by a patient during an ophthalmic examination or treatment procedure. The study room was 10 m (L) × 10 m (W) × 5 m (H), which is larger than any ophthalmic consulting ward, and was ventilated by a combination of a ceiling air supply and a floor air exhaust. The ventilation rate was 3 ACH, and the velocity of the air supply was 0.0042 m/s, which is ~1/100th of the maximum velocity of the thermal plume of the human body; it therefore did not significantly affect the local microenvironment of the ophthalmologist and patient models.

**Figure 1:**
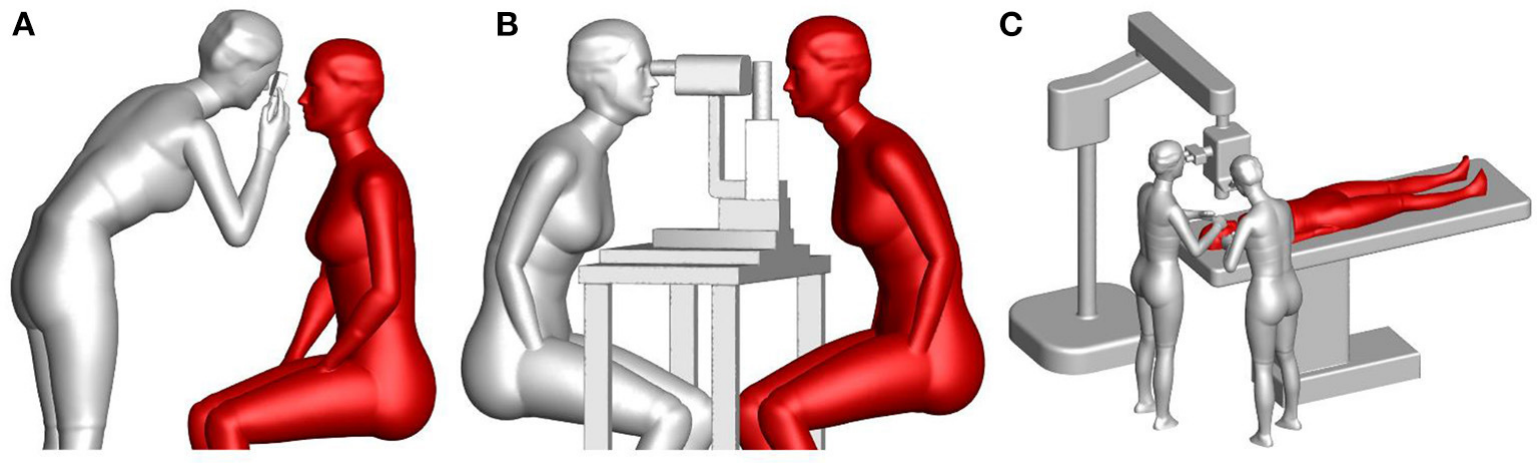
Numerical models of typical ophthalmic procedures: **(A)** a direct ophthalmoscopic examination, **(B)** a slit-lamp microscopic examination, and **(C)** an ophthalmic operation (gray manikin = ophthalmologist, red manikin = patient).

To accurately study the exposure of an ophthalmologist to droplets exhaled by a patient, accurate numerical models of breathing thermal manikins with adjustable body posture were generated to mimic the posture and relative position of ophthalmologists and patients in typical ophthalmic examination or treatment scenarios. These manikins had finely detailed faces and hands, and thus could model an ophthalmologists' inhalation exposure, mucous membrane exposure, and contact exposure to droplets exhaled by patients. Each manikin was 1.68 m tall and had a total skin-surface area of 1.45 m^2^, a mouth area of 1.6 cm^2^, a nostril cross-sectional area of 0.52 cm × 2.0 cm and an eye area of 1.75 cm × 2.0 cm. Siemens NX 10.0 was used to build three-dimensional models of an ophthalmoscope, a slit-lamp microscope, an operating microscope and a hospital bed. Then, the relative positions of the manikins and equipment models were adjusted to generate three typical ophthalmic examination or treatment scenarios: a direct ophthalmoscopic examination, a slit-lamp microscopic examination and an ophthalmic operation, as shown in Figure 1. In the ophthalmic operation scenario, the ophthalmologists were standing instead of sitting, which is uncommon. However, the distance between the breathing zones of the ophthalmologists and the patient was determined by the location of the microscope and the patient's bed, and thus the ophthalmologists' posture had little effect on their exposure to droplets exhaled by the patient. In addition, as the exhaled droplets are very small and not irritant therefore exhaled aerosol exposure is considered inert and not triggering any eye reactions like blinking or tearing during exposure.

### Simulation Setup

The computational fluid dynamics (CFD) numerical simulation required the computational domain to be divided into meshes. This was performed using the mesh settings described in Appendix A. The theoretical methods of the numerical simulation are given in Appendix B, and the boundary conditions and the setup for the simulation of human respiration and coughing are given in Appendix C.

To enable direct comparisons to be made between an ophthalmologist's exposure in each scenario, the same ambient parameters were used for all three scenarios: a ventilation rate of 3 ACH, an air velocity of 0.0042 m/s, an air temperature of 293 K and an RH of 40%. Ophthalmologists breathe through their nose, while patients tend to breathe through their mouth, due to nervousness. Thus, the temperature of the patient's exhaled airflow was 306 K, and the patient exhaled pathogenic droplets in the second cycle of breathing, but did not exhale droplets in the following breath cycle. The boundary conditions of droplets deposited on the skin surface of a thermal manikin were set as trap boundary conditions, while those of droplets inhaled by the nose and mouth were set as escape boundary conditions. Approximately 180 droplets were released in each time-step, which was 0.1 s during breathing. Calculations were performed for 320 time-steps, which spanned a total of 32 s, during which time ~10,000 exhalation parcels were released by the patient.

Coughing is a much more short-lived process than breathing, and thus the time-step in the first 0.6 s of coughing was set to 0.005 s, and 120 time-steps were calculated for this setting. The interval following coughing was set to 0.4 s, after which a normal breathing process with a 0.1 s time step resumed, and 320 time-steps were calculated for this setting. If coughing resulted in the exhalation of pathogenic droplets, breathing did not. Non-slip boundary conditions were used for all walls.

## Results

### Comparison of Exposure in Typical Ophthalmic Procedures

A steady-state airflow field was calculated for each ophthalmic examination or treatment scenario, and the result of each calculation was used as the initial value for each transient calculation. Then, transient boundary conditions were used for respiration, and droplets was simplified to spherical with an initial diameter of 5 μm were released from the patient's mouth. The behavior of these droplets was modeled and used to calculate the unstable flow field. To eliminate errors due to the random movement of droplets, which resulted from the application of a stochastic model for droplet tracking, the calculations for each numerical simulation condition were repeated three times. And then the average and standard deviation of the data are calculated statistically.

**Figure 2:**
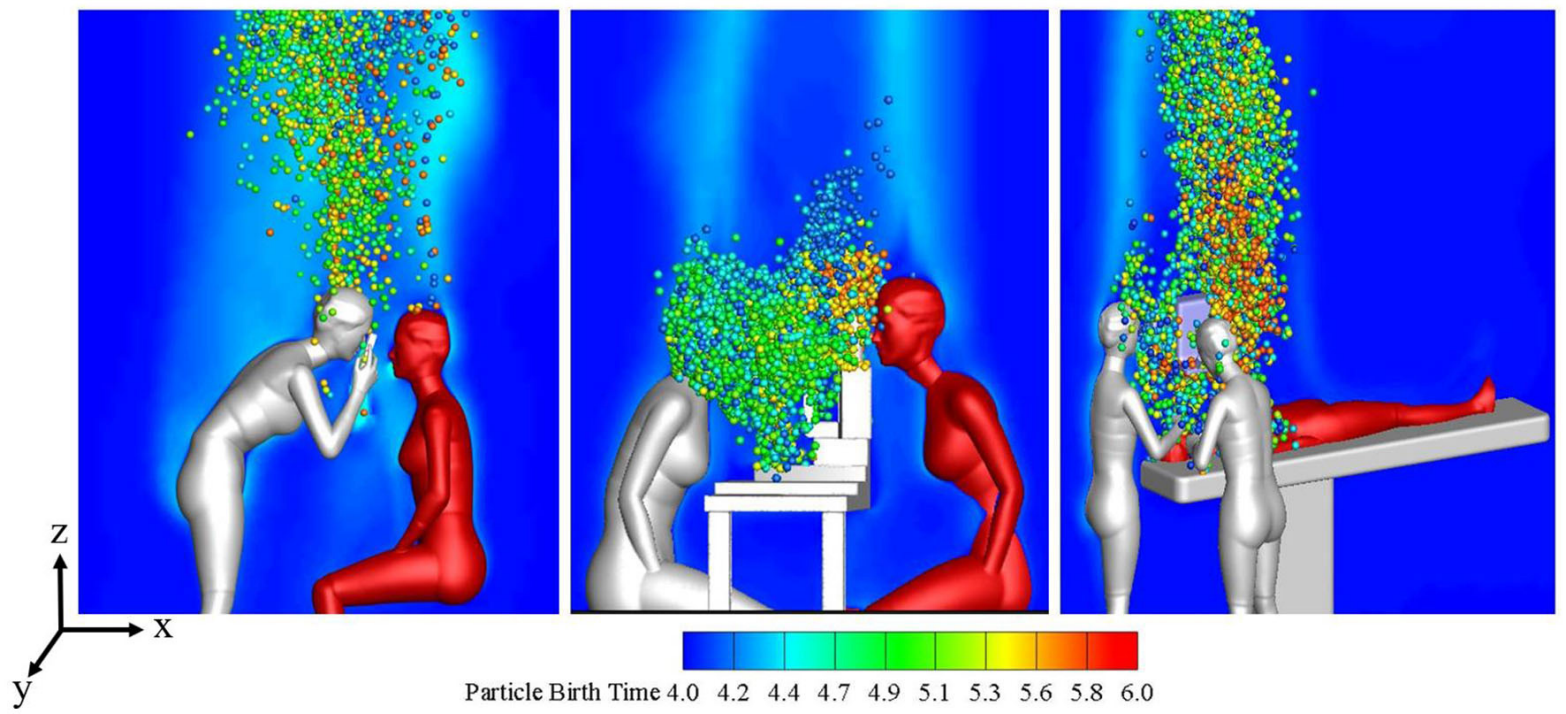
Flow patterns and droplet distribution on plane *y* = 0 at *t* = 16s during three ophthalmic examination or treatment scenarios.

Figure 2 shows the distribution of the local microenvironmental airflow and droplets on the surface plane of *y* = 0 at *t* = 16 s during the three typical ophthalmic examination or treatment scenarios. The CFD simulation results for the transient airflow and droplets in each scenario were analyzed to quantify the droplet exposures for ophthalmologists in these scenarios.

**Figure 3:**
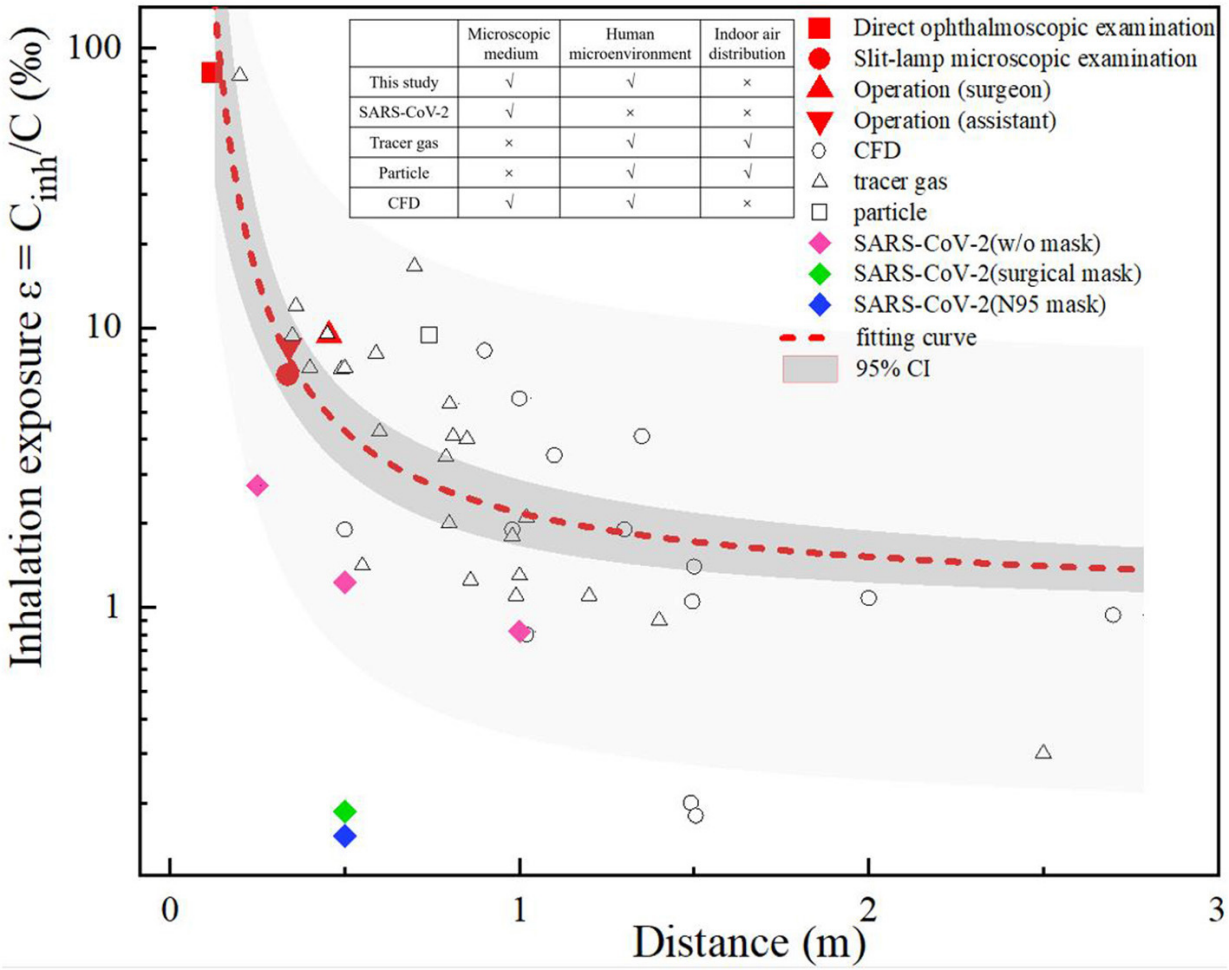
Inhalation exposure of ophthalmologists' compared to previous studies.

A comparison was made between the ophthalmologist's inhalation exposure in this study and the interpersonal exposure that has been reported in previous studies. As shown in Figure 3, the previous studies were classified as CFD (17–22) or experimental studies, where the latter used tracer gas (23–33) or particles (34) to simulate droplets or droplet nuclei. In addition, Ueki (35) compared the inhalation exposure to live SARS-CoV-2 particles when wearing no mask, a surgical mask or an N95 respirator. The inhalation exposure index is expressed as Cinh/C, where Cinh is the concentration of a contaminant inhaled by a susceptible individual and C is the concentration of a contaminant released from the source.

We set three biomimetic levels of human-to-human droplet transmission, namely a microscopic medium level (i.e., that which occurs in simulations of droplet nuclei and virus particles), a human microenvironmental level (that which occurs in thermal plume and breathing airflow) and an indoor air-distribution level. A comparison of previous studies in terms of these biomimetic levels is shown in Figure 3. Due to the different methods developed in these previous studies, the interactions between two people, the ventilation modes, the environmental conditions, and the diameter and the tracer modes of exhaled droplets varied in these studies, and thus there are differences between the data of these studies. However, the comparison shows overall trends, which indicated that when two people were close to each other (<1 m apart), their inhalation exposure decreased with interpersonal distance, the overall variance in their inhalation exposure was large, and the interaction of the microenvironment between them was the dominant factor affecting their inhalation exposure. In contrast, when two people were separated by a distance >1 m, the overall variance in their inhalation exposure was small, and the room airflow was the dominant factor affecting their inhalation exposure.

During the direct ophthalmoscopic examination scenario in this current study, the distance between the mouth of the ophthalmologist and the patient was only 0.12 m; in this setting, the ophthalmologist's inhalation exposure was 95 times that of a person involved a normal human interaction at a distance of 1 m, and was greater than that in all previous studies (a distance of >0.2 m). During the slit-lamp microscopic examination scenario, the distance between the ophthalmologist and the patient was also short (0.34 m), but as the slit-lamp microscope acted as a barrier, the exposure of the ophthalmologist was approximately the same as that of an ophthalmologist positioned 0.6 m away from a patient, as in previous studies (24, 34). During the ophthalmic surgery scenario, the distance between the ophthalmologists and the patient was ~0.35–0.45 m, and the ophthalmologists' inhalation exposure was slightly higher than that during the slit-lamp microscopic examination scenario. As mentioned above, the distance between the eyepiece of the operating microscope and the patient's eye was constant during this ophthalmic surgery scenario, and thus the microenvironment between the ophthalmologists and the patient was unaffected by the posture (i.e., sitting or standing) of the former.

**Figure 4:**
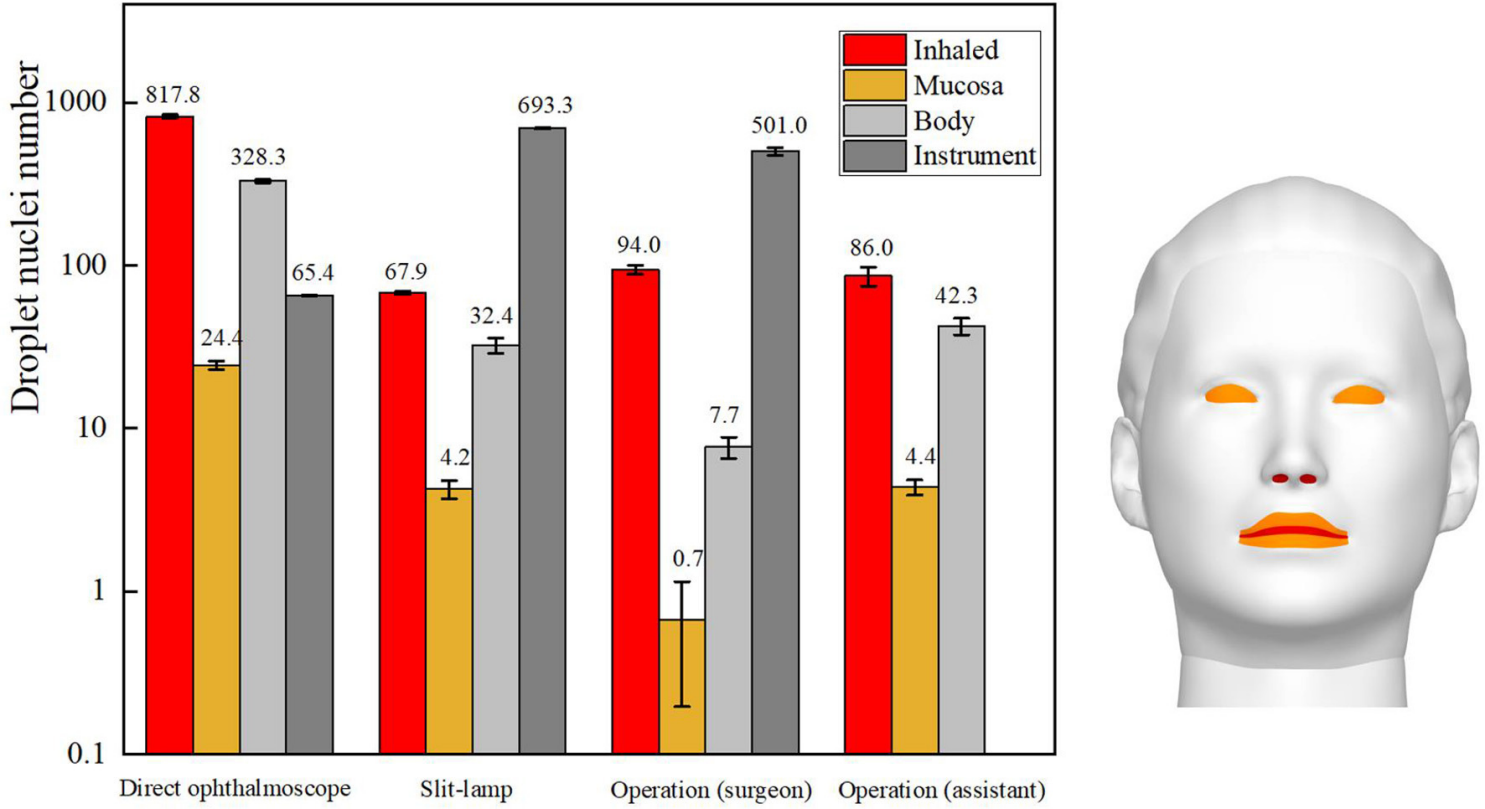
Sites of ophthalmologists' exposure during three ophthalmic examination or treatment scenarios.

The total number of droplets released by patients in each ophthalmic examination or treatment scenario was calculated to be 10,000, and the droplet exposure for each route of transmission was quantified by counting the number of droplets or droplet nuclei inhaled by or settled on the surfaces of ophthalmologists or settled on instruments. The number of droplet nuclei to which ophthalmologists were found to be exposed by each transmission route during each ophthalmic examination or treatment scenario is shown in Figure 4. The results showed that the greatest inhalation exposure occurred during the direct ophthalmoscopic examination scenario, as in this scenario the ophthalmologist inhaled 12.1 times the number of droplet nuclei inhaled by the ophthalmologist during the slit-lamp microscopic examination scenario, 8.8 times the number inhaled by the surgeon during the ophthalmic surgery scenario and 9.7 times the number inhaled by the assistant during the ophthalmic surgery scenario. The mucosal deposition exposure of the ophthalmologist was also highest during the direct ophthalmoscopic examination scenario, and was 5.5 times the mucosal deposition exposure of the ophthalmologist during the slit-lamp microscopic examination scenario and 6.3 times the mucosal deposition exposure of the assistant during the ophthalmic surgery scenario (the surgeon did not have any mucosal deposition exposure during the ophthalmic surgery scenario). Finally, the greatest number of droplets were deposited on the surface of the slit-lamp microscope during the slit-lamp microscopic examination scenario, while the greatest number of droplets settled on the ophthalmologist's body surface during the direct ophthalmoscopic examination scenario.

In summary, the inhalation and mucosal exposure of the ophthalmologist were greatest during the direct ophthalmoscopic examination scenario. This is attributable to the operational requirements of an ophthalmoscope, which necessitate a relatively short distance (~12 cm) between an ophthalmologist and a patient. The small size of the hand-held ophthalmoscope (~15 cm × 5 cm) meant that a relatively small number of droplets were deposited on the ophthalmoscope during the direct ophthalmoscopic examination scenario. The inhalation and indirect exposure of the ophthalmologist during the slit-lamp microscopic examination scenario was slightly higher than that of the ophthalmologist during the ophthalmic surgery scenario, whereas the largest number of droplets were deposited on the slit-lamp microscope during the slit-lamp microscopic examination scenario. As the skin of a patient is in close contact with this instrument during an examination, this represents a significantly increased risk of indirect exposure for an ophthalmologist.

### Comparison of Exposure From Breathing vs. Coughing

**Figure 5:**
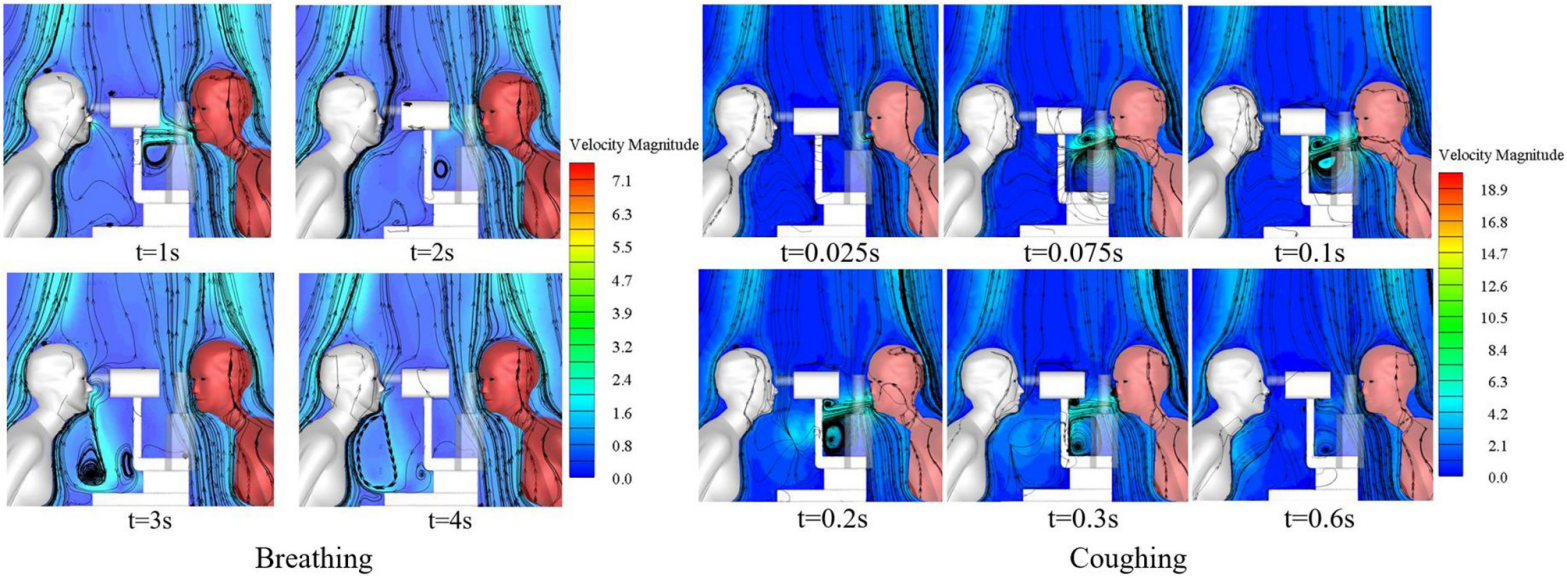
Variation of microenvironmental airflow with time.

**Figure 6:**
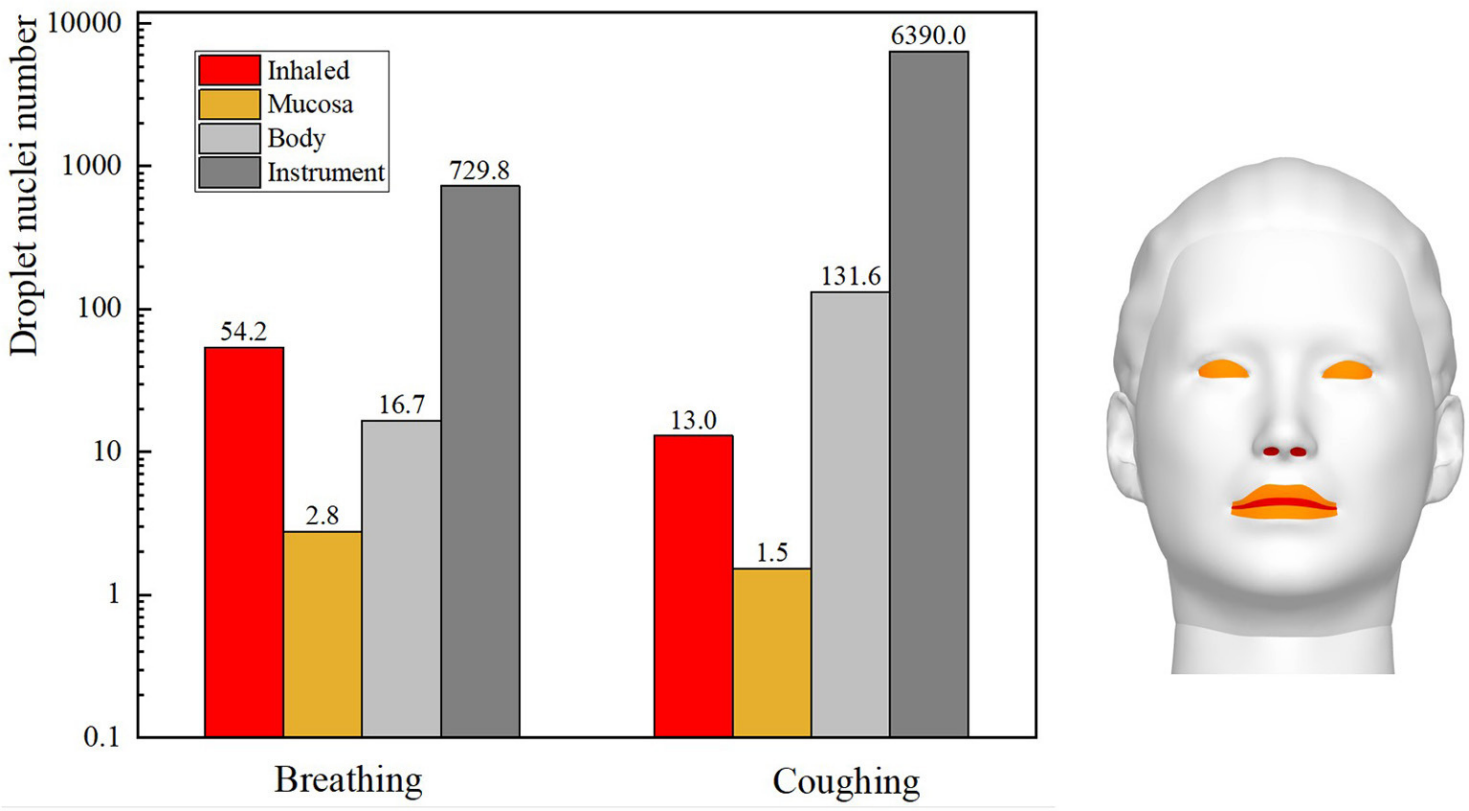
Exposure of an ophthalmologist to droplets released by a patient in different exhalation patterns.

The slit-lamp microscopic examination scenario is the most common type of ophthalmic examination scenario and was therefore used to compare the exposure of ophthalmologists to droplets released as the patient breathed with their exposure to droplets released as the patient coughed. The single coughing process of the patient lasted for 0.6 s, and was followed by respiration with a phase difference of π. The initial particle size of the cough-exhaled droplets was 50 μm, whereas the initial particle size of the respiratory-exhaled droplets was 10 μm. A comparison between the airflow of a single cycle of breathing and that of a single cycle of coughing at different times is shown in Figure 5, and the droplet exposures from these two cycles *via* different routes are shown in Figure 6.

During coughing, the peak velocity of the airflow from a person's mouth can reach 20 m/s. The results showed that the ophthalmologist inhaled 54.2 and 13 droplets of every 10,000 droplets in a patient's exhaled breath and cough, respectively. However, the number of droplets released by a person's cough is ~30 times the number of droplets released in a person's breath, and thus the direct exposure to the ophthalmologist when the patient coughed was ~7.6 times that when the patient breathed. The initial size and the Stokes number of cough-exhaled droplets were greater than those of breath-exhaled droplets, as the gravity and inertia forces affecting droplets in a cough-exhalation are greater than those affecting droplets in a breath-exhalation; thus, the cough-exhaled particles had lower fluidity in the airflow. In addition, the slit-lamp microscope acted as a barrier between the ophthalmologist and the patient, and thus the greatest proportion of high-momentum large droplets settled on the surface of the slit-lamp microscope.

### Effect of Environmental RH on Exposure

Many studies have shown that RH has a significant effect on the evaporation time of droplets (29, 36). A high RH was shown to significantly delay the evaporation of large droplets (≥ 30 μm) exhaled by coughs because the driving force of mass transfer is the difference between the partial pressure of water vapor and air, and moist air absorbs less water vapor than dry air. Thus, the slit-lamp microscopic examination scenario was used to study the evaporation and distribution of 50-μm droplets cough-exhaled by the patient and the exposure of the ophthalmologist to these droplets. After coughing, the patient resumed breathing, with a phase difference of π.

**Figure 7:**
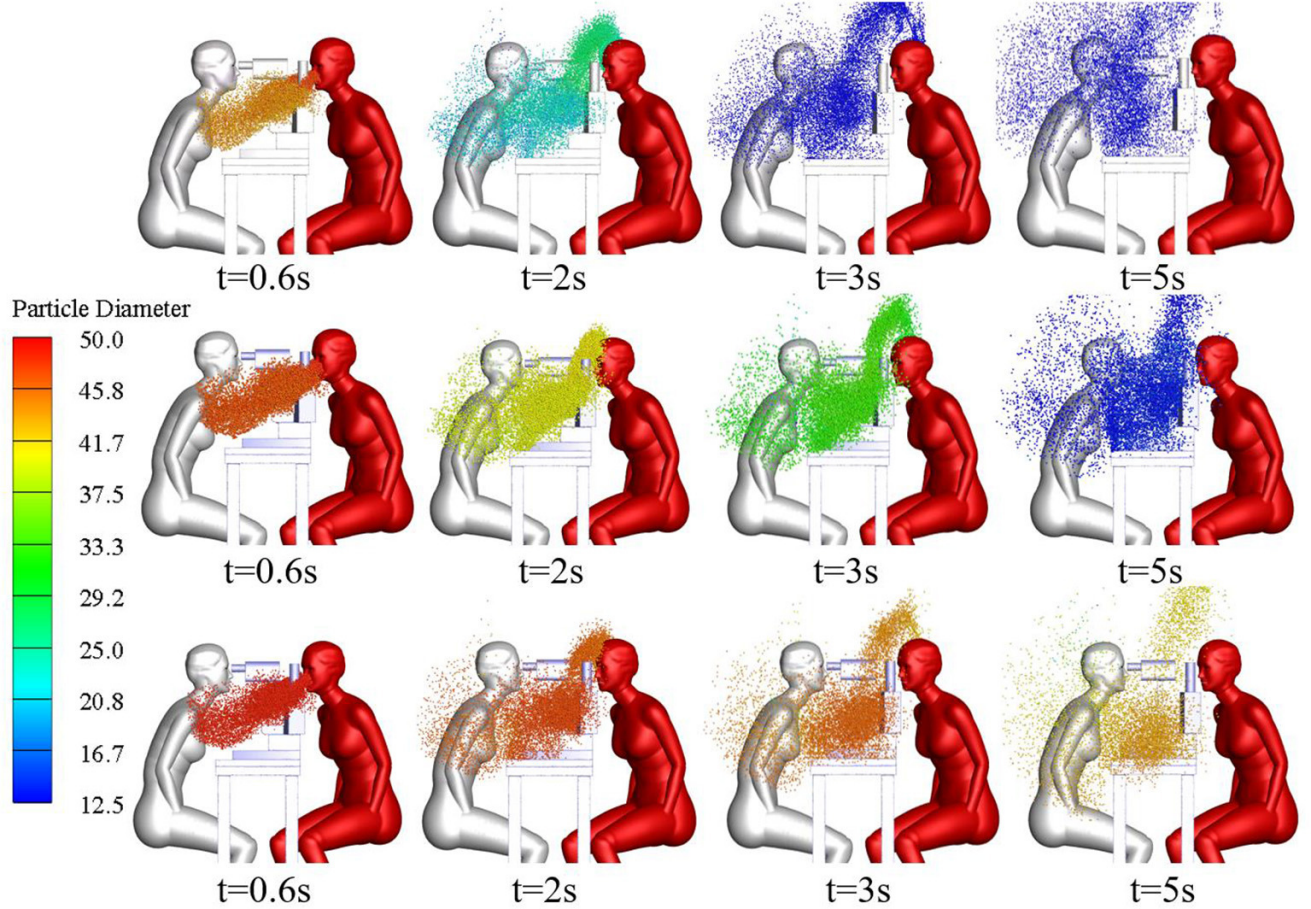
Comparison of cloud images of droplet-size distribution when the RH is 40, 70, or 95%.

**Figure 8:**
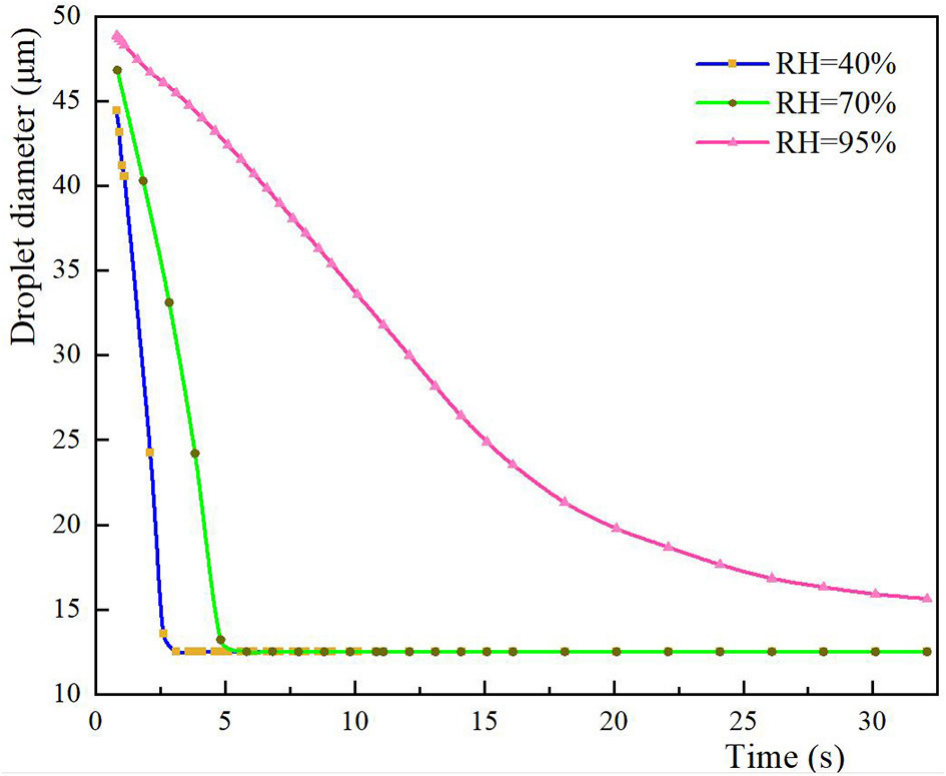
Comparison of droplet evaporation times when the RH is 40, 70, or 95%.

The size and spatial distribution of exhaled droplets at different times and RH levels are shown in Figure 7, and a comparison of the evaporation time of cough-exhaled droplets at different RH levels is shown in Figure 8. The results showed that the evaporation time of droplets was delayed at increased RH levels. Specifically, when the RH was 40 or 70%, the droplets evaporated to droplet nuclei within 10.2 s, whereas when the RH was 95%, this time was longer than 32 s.

**Figure 9:**
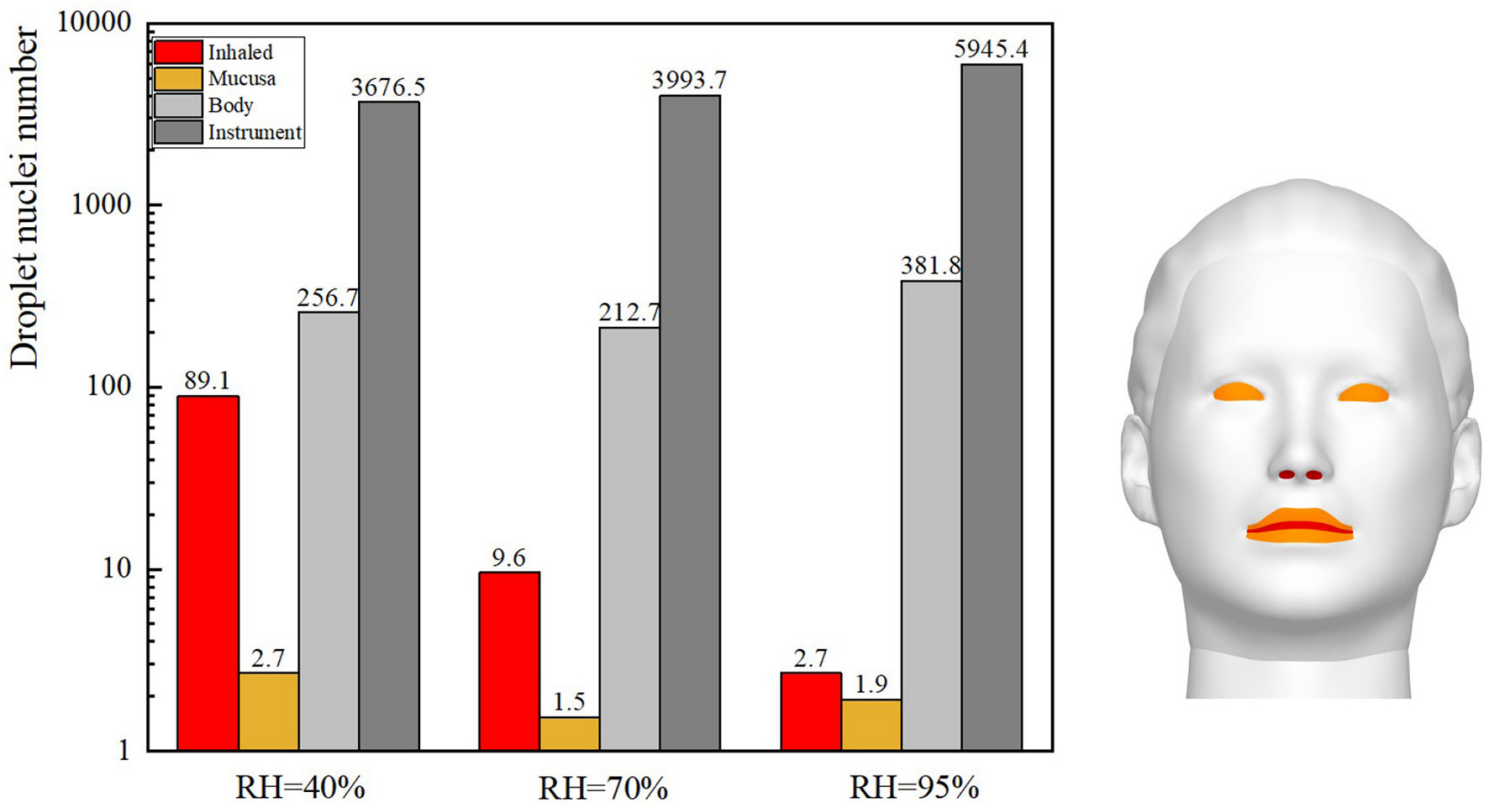
Droplet exposure of ophthalmologists when the RH is 40, 70, or 95%.

The ophthalmologist's exposure to droplets released by the patient at various RH levels was statistically analyzed, as shown in Figure 9. As the RH was increased, the inhalation exposure of ophthalmologists decreased and the number of droplets deposited on the slit-lamp microscope increased. This indicates that when RH levels are high, this instrument must be regularly disinfected. The ophthalmologist's mucosal deposition and indirect exposure were lowest when the RH was 70%. In addition, at high RH levels, the ophthalmologist's direct and indirect exposure were low and high, respectively.

### Influence of Initial Size of Droplets on the Ophthalmologist's Droplet Exposure

**Figure 10:**
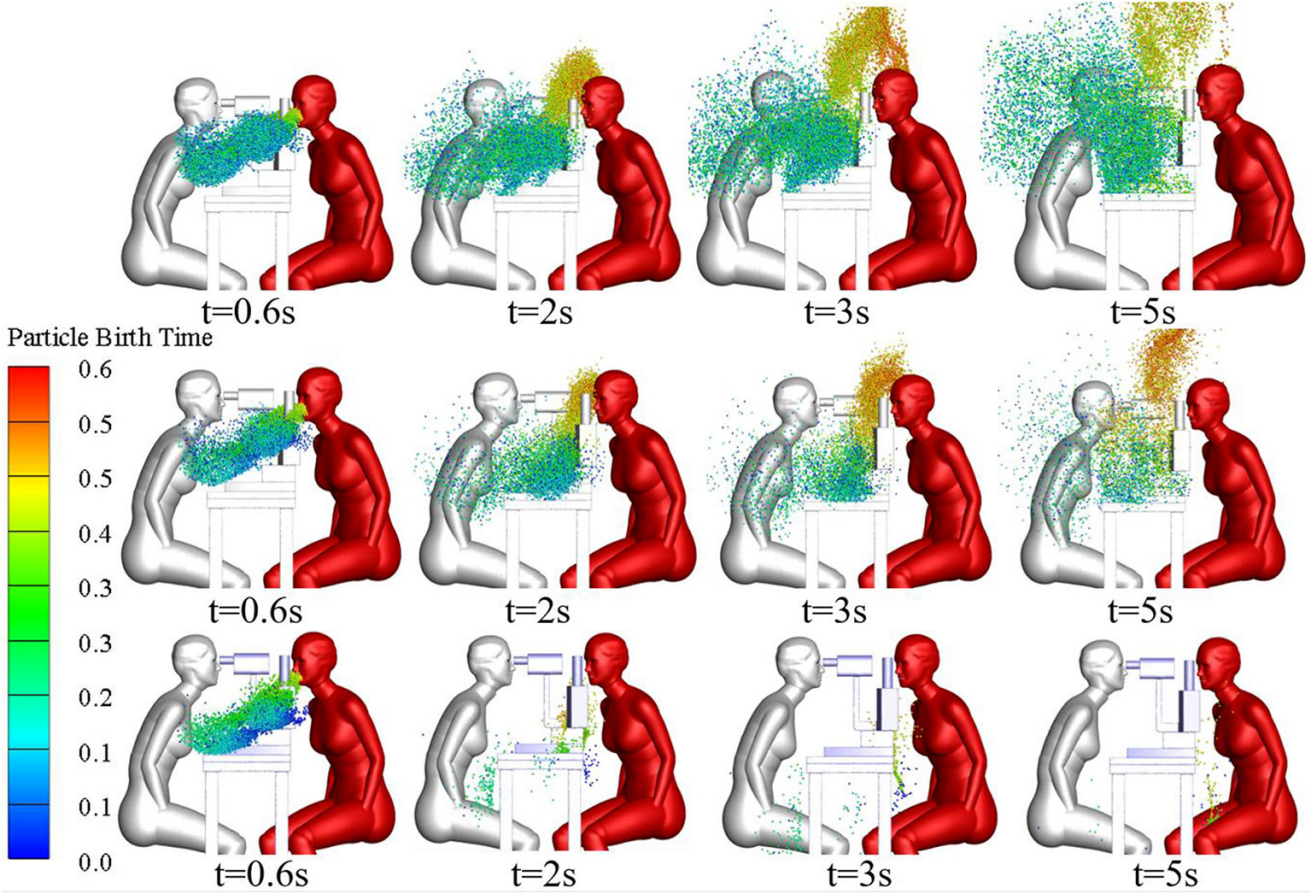
Comparison of droplet distributions with initial droplet diameters of 50, 70, or 100 μm.

**Figure 11:**
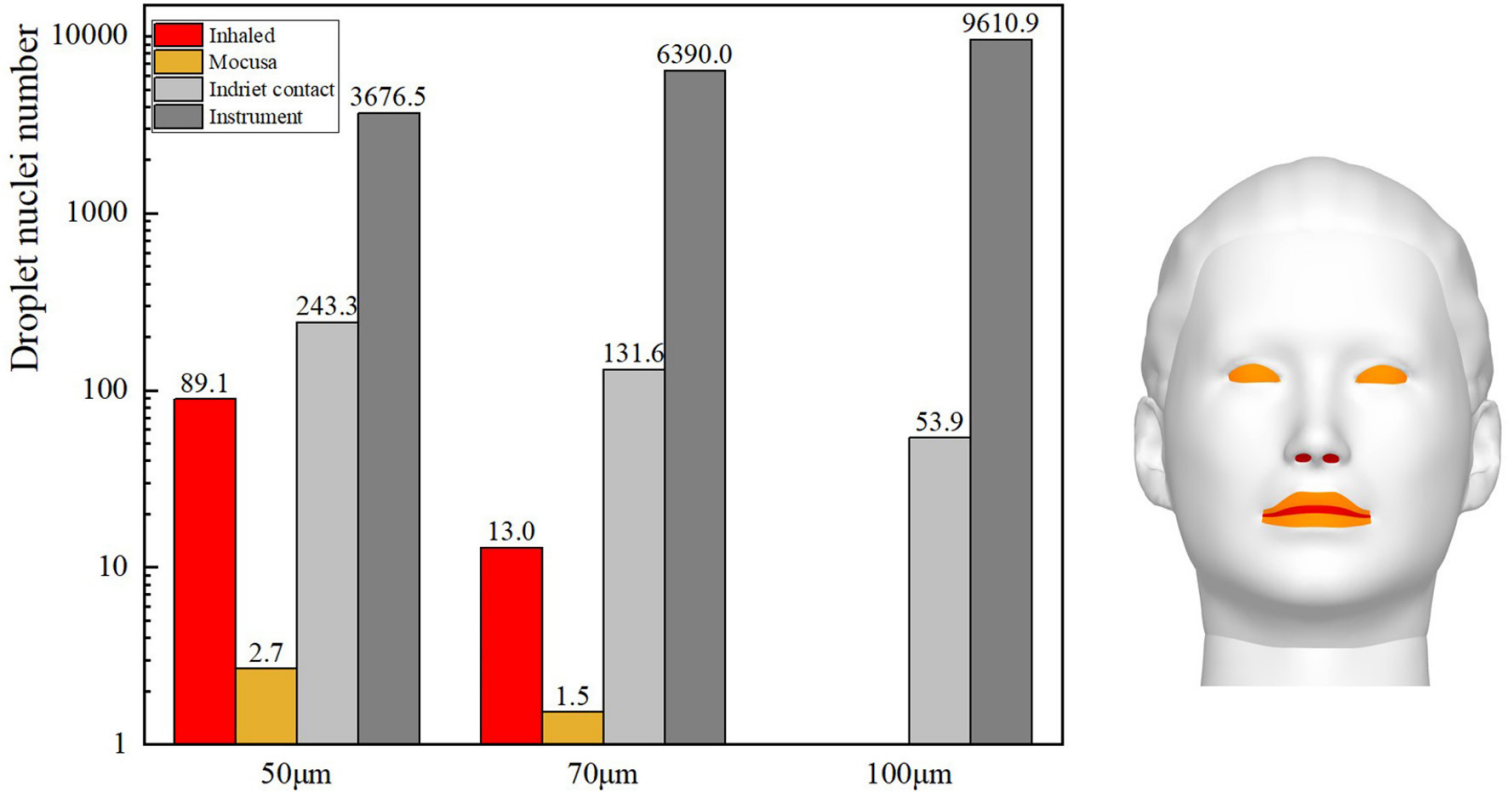
Exposure of ophthalmologists to initial droplets with diameters of 50, 70, or 100 μm.

Gravity has been shown to play a key role in the process of droplet dispersion (37–41). In this study, the distribution of cough-exhaled droplets with an initial diameter of 50, 70, or 100 μm was examined, and the ophthalmologist's exposure to these was determined. Cloud images of the results are shown in Figure 10, and a comparison of the ophthalmologist's exposure is shown in Figure 11.

The results show that the ophthalmologist's inhalation exposure to cough-exhaled droplets and the number of these droplets deposited on the ophthalmologist's mucosa and body decreased as the initial size of the droplets was increased. In addition, the number of droplets deposited on the slit-lamp microscope increased as the initial size of the droplets was increased, indicating that these instruments must be regularly disinfected. However, the largest droplets (100 μm) fell to the floor most rapidly, and thus few of these droplets were inhaled by the ophthalmologist. Finally, as the droplet diameter was increased, there was a decrease in the ophthalmologist's direct exposure and an increase in the ophthalmologist's indirect exposure.

## Discussion

The latest study suggests that patients with COVID-19 who have no symptoms or mild symptoms can have the same level of ability to transmit the virus as those who are sick enough to require hospitalization, and more than a third of those with very high viral loads had no symptoms or very mild symptoms (42). Besides short-airborne droplet inhalation, mucosa deposition and indirect contact (2), additional routes for SARS-CoV-2 transmission are also observed, such as fecal-oral transmission (43), conjunctival transmission (44), blood-borne transmission (45), and sexual transmission (46). It thus appears likely that SARS-CoV-2 can enter the human body *via* mucosal surfaces in areas such as the respiratory tract, the conjunctiva and the nose. Moreover, many studies have found direct and indirect evidence of SARS-CoV-2 transmission *via* the ocular route. For example, studies have detected SARS-CoV-2 RNA in the tears or conjunctival secretions of patients infected with SARS-CoV-2 (44, 45, 47–49). Furthermore, the wearing of eye glasses was shown to provide effective protection against SARS-CoV-2 infection, especially in high-risk situations (50). SARS-CoV-2 was also detected on the environmental surfaces of an ophthalmology examination room after visits by patients (51), and other studies have confirmed that SARS-CoV-2 can survive for days on dry surfaces (52, 53). However, real-time polymerase chain reaction could only detect viral material, not the infectivity of these virus samples. These findings indicate that ophthalmologists have a relatively higher risk of exposure to SARS-CoV-2 than some other medical professionals, and that they must use effective protective equipment and procedures in their clinical practices during the pandemic (54). This has been emphasized by professional organizations such as the Chinese Medical Association of Ophthalmology and the American Academy of Ophthalmology, who have recently warned that ophthalmologists must wear ocular protection when examining patients during the pandemic (35, 55). Thus, ophthalmologists must protect themselves from SARS-CoV-2 infection, and would be assisted to do so if they could quantify the risk of SARS-CoV-2 transmission in their practices.

Slight differences can occur by employed a Gaussian distributed random velocity fluctuation in the Discrete Random Walk model. Therefore, three independent calculations were conducted in each situation and then obtained convincing conclusions through statistical analysis. The calculation results revealed that during the three examination or treatment scenarios, the ophthalmologists had varying levels of direct inhalation and mucosal exposure and indirect exposure due to surface deposition of particles on their body and on instruments. These exposures may result in ophthalmologists becoming infected with SARS-CoV-2, and such infection could spread within an ophthalmology department and thereby accelerate the risk of pandemic spread. Therefore, ophthalmologists and related medical staff in an ophthalmologic department must use effective personal protection against droplet exposure during direct ophthalmoscopic examinations, slit-lamp microscopic examinations or ophthalmologic operations. However, as HCWs must work long shifts (56) amid a scarcity of equipment, a lack of knowledge and a low perception of risk (57), their level of compliance with such standard precautions is low (58, 59).

However, as a main route of transmission of SARS-CoV-2 involves the entry of pathogenic droplets into the human respiratory system, wearing a surgical mask or N95 respirator can significantly reduce the risk of SARS-CoV-2 infection due to droplet inhalation (42, 60, 61). The American Academy of Ophthalmology has recommended the use of slit-lamp microscope barriers or breath shields as an added measure of protection during slit-lamp microscopic examinations. However, the use of these breath shields does not completely block the passage of droplets into an ophthalmologist's area (62). Thus, protective suits, disposable gloves (60), goggles or a face shield (63), and a protective cap must also be worn to prevent indirect exposure to pathogen droplets settled on the surface of human skin or clothing.

If ophthalmologists take adequate precautions during ophthalmic examinations and treatments, such as by wearing masks and protective clothing, an operating cap (if required) and gloves, their inhalation and indirect droplet exposure could be effectively reduced. However, as the calculations showed that droplets deposited on the exposed eyes and facial skin of the assistant during the ophthalmic operation scenario, goggles should also be worn in this setting to prevent SARS-CoV-2 infection. Moreover, many droplets or droplet nuclei containing SARS-CoV-2 settled on the surfaces of instruments used during the ophthalmological examination scenario, and thus these instruments must be fully disinfected before and after use to ensure that indirect transmission does not occur.

In addition to the above recommendations, exposure to droplet nuclei suspended in the air of an ophthalmology department could be eliminated by using air purification devices, increasing the number of air changes per hour and using a negative air flow (60, 61). Moreover, ophthalmologists must make facial contact with the eyepiece of optical microscopes during many ophthalmic examinations or treatments. Eye PPE cannot be used in this situation, as it can obscure ophthalmologists' vision and lead to less accurate diagnosis of patients' conditions and medical errors during ophthalmic surgery. Therefore, medical equipment companies should be asked to develop new products to protect the eyes and faces of ophthalmologists, thus enabling ophthalmologists to provide accurate and high-quality medical services to patients while also protecting themselves from SARS-CoV-2 infection. Any new protective products must be fully validated to ensure that they effectively reduce droplet exposure. Normal social interactions, such as those occurring during transportation and family interactions, can also pose a risk of SARS-CoV-2 infection to HCWs (64, 65), so ophthalmologists must use PPE as necessary when in public or at home.

## Conclusion

Ophthalmologists need to be physically close to patients during ophthalmic examinations or treatment, and the relatively fixed near-distance relationship becomes a good model to study exposure risk of aerosol pathogens like SARS-CoV-2 between physician-patients. In this study, we have built models to simulate the most common scenarios in ophthalmological practice and studied the exposure of ophthalmologists to droplets exhaled by patients. The models have breathing thermal manikins and adjustable postures of patient and ophthalmologist to numerically simulate real world working condition. The results revealed that under direct ophthalmoscopy examination estimated aerosol exposure between the ophthalmologist and patient was 95 times higher than that of normal interpersonal interaction at a distance of 1 m. This aerosol exposure level of ophthalmologist to patient exhalation can further increase 7.6 times when the patient coughing during the examination. The exposure to deposited droplets on facility was high during the slit-lamp microscopic examination. The initial droplet sizes are related to exposure pattern, small size droplets cause more direct inhalation exposure, bigger droplets have more deposited on the examination instrument as expected. This study for the first time simulated the exposure risk of aerosol pathogens such as SARS-CoV-2 between physicians and patients, and identified the risk zone for strengthening protection during pandemic period.

## Data Availability Statement

The raw data supporting the conclusions of this article will be made available by the authors, without undue reservation.

## Author Contributions

YF and LL planned and conducted the research. All authors contributed to the manuscript writing.

## Funding

This work was supported by the National Key Research and Development Program of China (Grant No. 2020YFC0861500) and the National Natural Science Foundation of China (Grant No. 51778520).

## Conflict of Interest

HZ is employed by YuanPu EyePro Biopharm Limited, Chengdu, China. The remaining authors declare that the research was conducted in the absence of any commercial or financial relationships that could be construed as a potential conflict of interest.

## Publisher's Note

All claims expressed in this article are solely those of the authors and do not necessarily represent those of their affiliated organizations, or those of the publisher, the editors and the reviewers. Any product that may be evaluated in this article, or claim that may be made by its manufacturer, is not guaranteed or endorsed by the publisher.
