# Supplementary material for: Exposure of Ophthalmologists to Patients' Exhaled Droplets in Clinical Practice: A Numerical Simulation of SARS-CoV-2 Exposure Risk
Source: Front Public Health. 2021 Sep 20;9:725648. doi: 10.3389/fpubh.2021.725648 (PMC8488202; doi:10.3389/fpubh.2021.725648)
Supplement: Supplementary file 4 [file Data_Sheet_1.DOCX]

Supplementary Material

# Appendix A. Computational mesh

Unstructured hybrid poly-hexcore–prism meshes were generated using ANSYS Fluent Meshing 2020 R1. Eight prism layers were created near thermal manikin surfaces to contain the viscous sub-layers and enable near-wall gradients to be calculated accurately. In addition, the body meshes around the manikins and in the respiratory area between the ophthalmologist and the patient were encrypted to varying degrees. Mesh details are shown in Supplementary Figure 1. The y^+^ value of the thickness of the first prism layer was guaranteed to capture the laminar-to-turbulence transition sites using the transitional shear-stress transport (SST) model, where y^+^ was a dimensionless distance to a wall. The mesh topology was determined by refining the mesh until the flow field solutions achieved grid-independence. The final meshes of the computational domains for the direct ophthalmoscopic examination scenario, the slit-lamp microscopic examination scenario and the ophthalmic operation scenario contained 1,480,000 elements, 1,510,000 elements and 2,290,000 elements, respectively.

# Appendix B. Construction of the computational model

The well-validated Euler-Lagrange based multiphase flow model was used for CFD studies of the local airflow between the ophthalmologist(s) and the patient and the evaporation/diffusion of pathogenic droplets.

## Continuous phase (air flow)

Indoor airflow can typically be predicted by large eddy simulation (LES) and Reynolds average Navier-Stokes turbulence models. As the application of LES models requires enormous computational resources and long periods of time, the SST turbulence model was used instead because of its high computational efficiency and robustness. In addition, the SST model accurately predicts the k-ε model and k-ω model in a turbulent core region and near a wall, respectively and can effectively predict the laminar-to-turbulence transition.

Based on the Eulerian descriptions, the indoor airflow field can be predicted by solving the conservation laws. The governing equations are given as follows:

$$\frac{\partial u_{i}}{\partial x_{i}}=0$$

$$\frac{\partial u_{i}}{\partial t}+u_{j}\frac{\partial u_{i}}{\partial x_{i}}=-\frac{1}{\rho}\frac{\partial p}{\partial x_{i}}+\frac{1}{\rho}\frac{\partial\tau_{ij}}{\partial x_{i}}+g_{i}$$

where $\boldsymbol{u}_{\boldsymbol{i}}$ and $u_{j}$ (i, j = 1, 2 or 3) are the velocity components in the x, y and z directions; $\boldsymbol{p}$ is the pressure; and $\boldsymbol{g}_{\boldsymbol{i}}$= 9.81 m^2^/s is gravity, aligned with the negative z-direction.

In Eq. (2), the viscous stress tensor $\boldsymbol{\tau}_{\boldsymbol{ij}}$is defined as:

$$\tau_{ij}=\mu\left[ \left( \frac{\partial u_{i}}{\partial x_{j}}+\frac{\partial u_{j}}{\partial x_{i}} \right)-\frac{2}{3}\sigma_{ij}\frac{\partial u_{k}}{\partial x_{k}} \right]$$

The semi-implicit method for pressure-linked equations algorithm was used to compute the pressure–velocity coupling. The second-order upwind scheme was used to discretise the convection and diffusion-convection terms in the governing equation. In addition, the Boussinesq model was used to consider the buoyancy effect, in which air density was regarded as a constant except in the momentum equation of vertical velocity.

## Discrete phase (exhaled droplets and nuclei)

The discrete-phase model included in the ANSYS Fluent software was used to simulate the transmission of exhaled droplets by the patient. The particle trajectory was predicted by solving the following equations:

$$\frac{du_{p}}{dt}=F_{D}\left( \vec{u}-\vec{u}_{p} \right)+\frac{\vec{g}\left( \rho_{p}-\rho\right)}{\rho_{p}}+\vec{F}$$

where $\vec{u}$ is the fluid velocity, $\vec{u}_{p}$ is the particle velocity, $\rho$ is the fluid density, $\rho_{p}$ is the particle density, $\vec{F}$ represents the additional forces exerted on the particle, $F_{D}\left( \vec{u}-\vec{u_{p}} \right)$ is the drag force per unit particle mass and is calculated by:

$$F_{D}=\frac{18\mu}{\rho_{p}d_{p}^{2}}\frac{C_{D}{Re}_{p}}{24}$$

In the above equation, $\mu$ is the molecular viscosity of the fluid, $d_{p}$ is the particle diameter and ${Re}_{p}$ is the particle Reynolds number, which is defined as:

$${Re}_{p}\equiv\frac{{\rho d}_{p}\left| \vec{u}-\vec{u}_{p} \right|}{\mu}$$

In Eq. (4), the additional forces $\vec{F}$ comprise thermophoretic force, virtual mass force, pressure gradient force, Basset force, Brownian force, Saffman’s lift force and Magnus lift force. However, only thermophoretic force and Saffman’s lift force were considered in our study, as they played the most important role in particle motion near manikin skins, whereas the other forces were sufficiently small that their effects could be ignored.

An exhaled droplet consisted of water, and thus its density was set as 1,000 kg/m^3^ and its specific heat capacity as 4,182 kJ/kg·K. The volumetric ratio of volatile components to non-volatile components was 3:1.

To accurately simulate the process of droplet evaporation, the Keenan/Keyes equation was used to correct the saturation vapor pressure on the surface of droplets with respect to temperature change, and the average error of the equation was only 0.08 %.

# Appendix C. Boundary conditions

The influences of human heat flux at the body surface and exhalation flow were taken into consideration. In a previous study, the total heat of a human was determined to be 75 W, and thus the convection heat flux at the body surface was defined as 25 W/m^2^, as the surface area of manikin skin was 1.45 m^2^. The curve of human respiratory flow over time can be fitted with a sinusoidal function. The velocity boundary condition of a manikin breathing via its mouth or nose can be expressed as:

$$u=u_{max}\sin\omega\cdot t$$

The total amount of exhalation or inhalation in a single respiratory cycle can be expressed as:

$$Q_{T}=\int_{0}^{T/2} \left( Q_{max}\sin\omega\cdot t \right)dt$$

while $T=60/BF$ and $\omega=2\pi/T=\pi BF/30$

so, $Q_{max}=\pi RMV/60=u_{max}A$ $u_{max}= \pi RMV/60A$

In sum, the velocity boundary equation can be defined as:

$u= \pi RMV/60A\frac{\pi RMV}{60A}\sin\left( \frac{\pi BF}{30}\cdot t \right)$

where *RMV* is the respiration minute volume, namely the volume of gas exhaled per minute (L/min); *BF* is the breathing frequency, namely the number of respiratory cycles per minute (min^-1^); and *A* is the cross-sectional area (m^2^) of air flow through the mouth or nostrils.

As the ophthalmologist was performing light work and the patient may have been nervous (due the examination itself or due to exposure to the light of the instruments), the *RMV*s of the ophthalmologist and the patient were 15 L/min and their BF was 15 min^-1^.

Coughing is a common symptom of most respiratory diseases, including COVID-19, and is a primary route by which respiratory viruses are transmitted. Previous research tested the cough flow of 25 volunteers and found that the curve of dimensionless flow rate vs dimensionless time during a cough process approximately followed the Gamma function distribution. It was also shown that the cough flow at different times is a function of the cough peak-flow rate (*CPFR*), the peak velocity time (*PVT*) and the cough expired volume (*CEV*), all of which are affected by the height and weight of a human. The function of cough can thus be defined as:

$$Q=\frac{a_{1}CPFR\left( t/PVT \right)^{b_{1}-1}exp\left( -\frac{t}{c_{1}PVT} \right)}{\Gamma\left( b_{1} \right)c_{1}^{b_{1}}} \left( t/PVT<1.2 \right)$$

$$Q=\frac{a_{1}CPFR\left( t/PVT \right)^{b_{1}-1}exp\left( -\frac{t}{c_{1}PVT} \right)}{\Gamma\left( b_{1} \right)c_{1}^{b_{1}}}+\frac{a_{2}CPFR\left( t/PVT-1.2 \right)^{b_{2}-1}exp\left( -\frac{t/PVT-1.2}{c_{2}} \right)}{\Gamma\left( b_{2} \right)c_{2}^{b_{2}}} \left( t/PVT>1.2 \right)$$

The calculation method of the World Health Organization was used to determine that the height of the thermal manikin was 1.68 m, and its corresponding standard weight was 58.8 kg. And *CPFR*, *CEV* and *PVT* were calculated to be 3.88 L/s, 0.834 L and 71 ms, respectively. The corresponding image of the transient cough-jet airflow waveform is shown in Supplementary Figure 2.

The transient boundary conditions, such as breathing airflow, cough-jet airflow and the corrected temperature with respect to the saturated vapor pressure, were set by a user-defined function compilation.

| 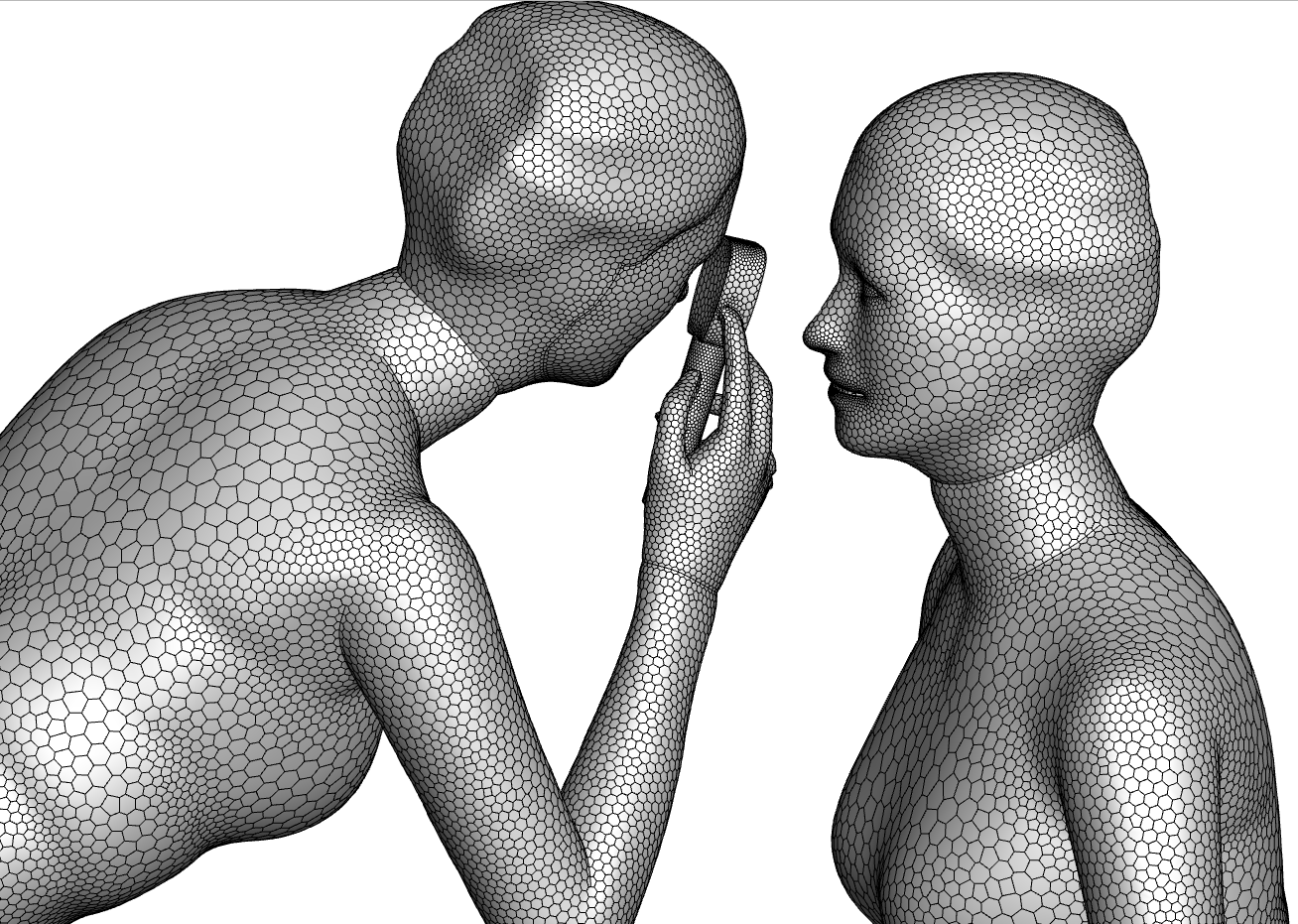 | 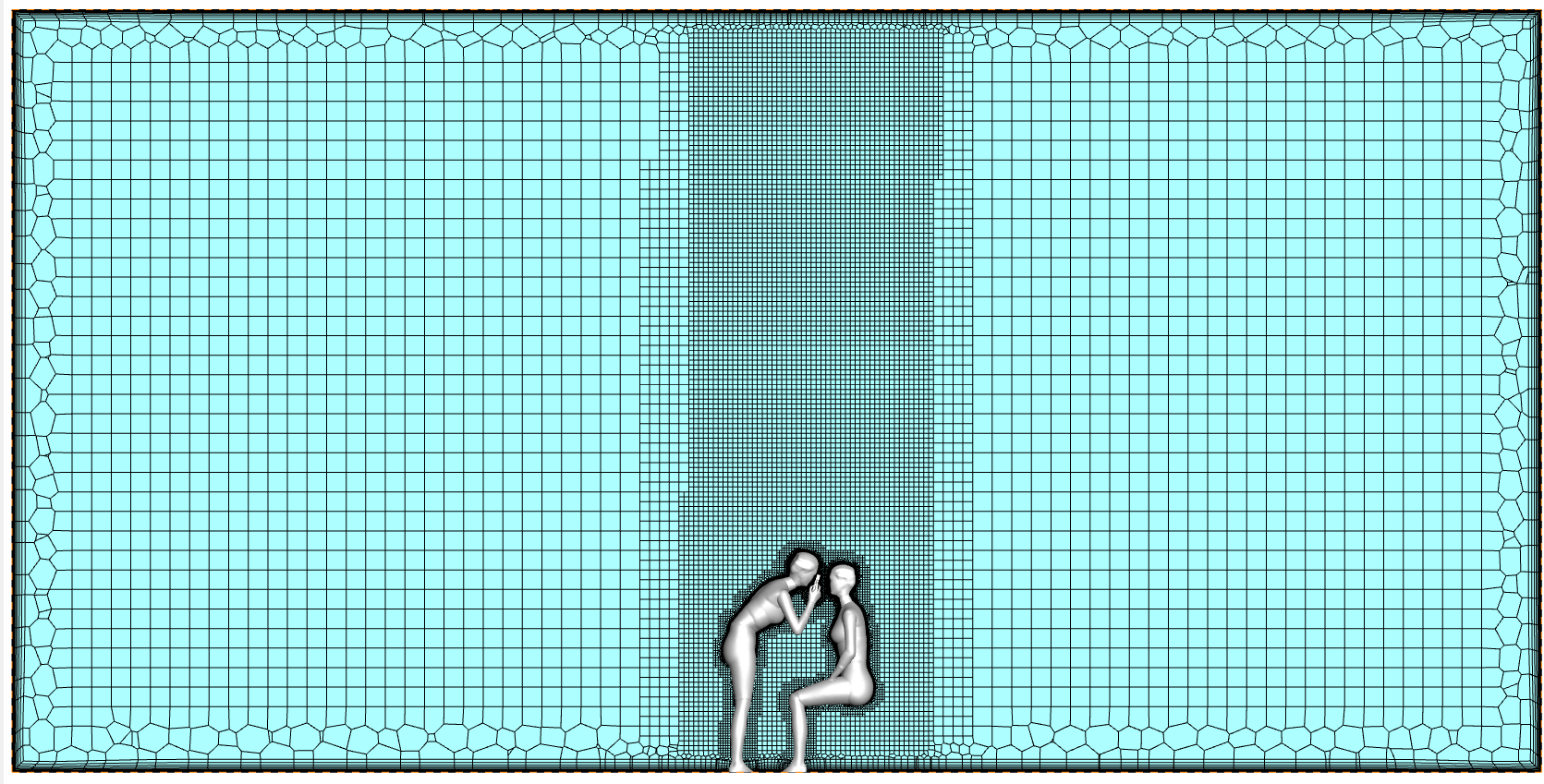 |
| --- | --- |
| (a) | (b) |
| 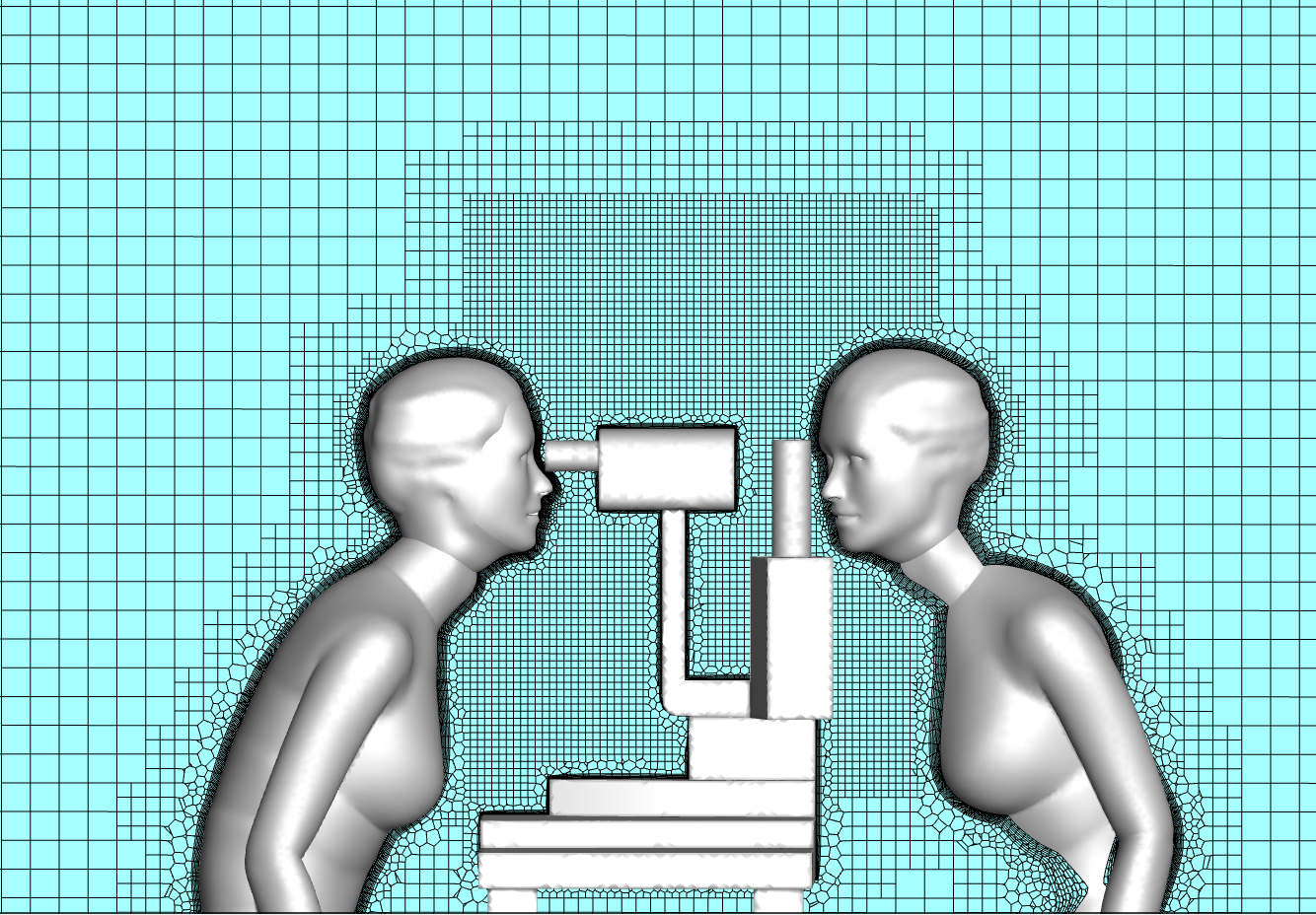 | 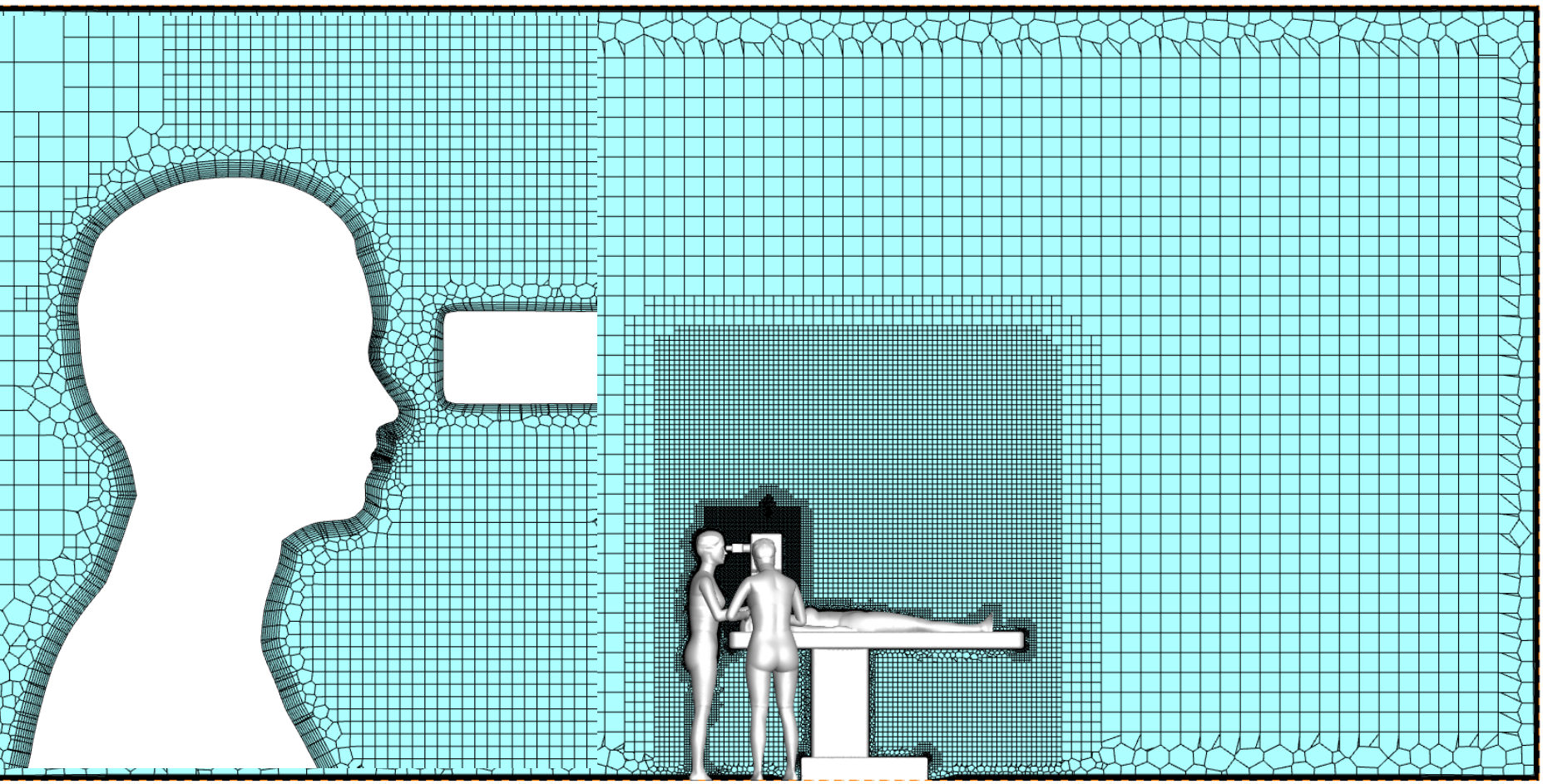 |
| (c) | (d) |
| **Supplementary Figure 1.** Mesh details of (a) the grid arrangements around the manikins, (b) the direct ophthalmoscopic examination scenario, (c) the slit-lamp microscopic examination scenario and (d) the ophthalmic operation scenario. | |

| 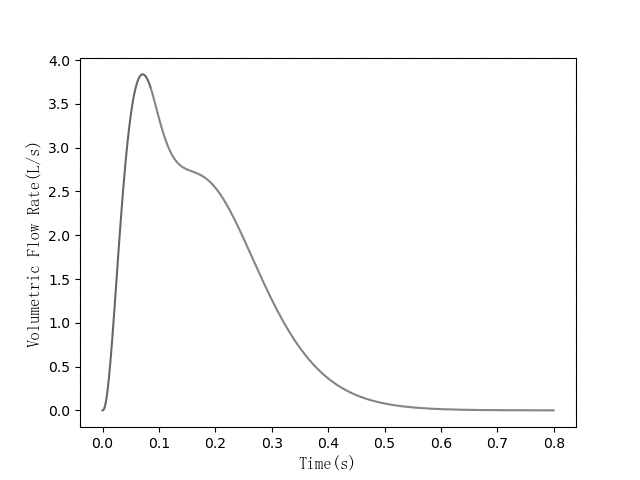 |
| --- |
| **Supplementary Figure 2**. Transient cough-jet airflow waveform |
